# Advances in theories and models of cardiac rehabilitation after acute myocardial infarction: A narrative review

**DOI:** 10.1097/MD.0000000000039755

**Published:** 2024-12-20

**Authors:** Jia Liu, Qiannan Zhao, Qianqian Shen, Xiaoqin Meng, Yi Zheng, Chen Lu, Yeping Zheng

**Affiliations:** a Department of Nursing, Second Affiliated Hospital of Jiaxing College, Jiaxing, Zhejiang Province, China; b Zhejiang Chinese Medical University, Hangzhou, Zhejiang Province, China; c Department of Cardiology, Second Affiliated Hospital of Jiaxing College, Jiaxing, Zhejiang Province, China; d Zhejiang University City College, HangZhou, Zhejiang Province, China.

**Keywords:** acute myocardial infarction, cardiac rehabilitation, model, theory

## Abstract

Acute myocardial infarction (AMI) remains a leading cause of mortality and disability worldwide, with its high mortality and recurrence rates significantly impacting patients’ quality of life and prognosis. Cardiac rehabilitation, as a comprehensive intervention strategy, plays a crucial role in improving outcomes for AMI patients. The aims to comprehensively analyze and evaluate the theoretical foundations and practical models of cardiac rehabilitation following AMI, providing healthcare professionals with up-to-date research advances and practical guidance. We systematically searched databases including PubMed, Web of Science, and Cochrane Library for relevant literature published between 2010 and 2023, selecting and analyzing high-quality studies related to theories and models of cardiac rehabilitation after AMI. This review elaborates on 7 major theories and models of cardiac rehabilitation, including the transtheoretical model, information-motivation-behavioral skills model, and self-determination theory. Analysis indicates that these theories and models have positive effects on improving patients’ quality of life, enhancing treatment adherence, and reducing rehospitalization rates. However, the applicability of different theories and models varies across cultural backgrounds and healthcare systems. Significant progress has been made in the research of theories and models for cardiac rehabilitation after AMI. However, further large-scale, multi-center studies are needed to validate their long-term effectiveness. Future research should focus on integrating these theories and models with emerging technologies to enhance the accessibility and efficacy of cardiac rehabilitation.

## 1. Introduction

Cardiovascular disease has become 1 of the foremost health threats globally, with both incidence and mortality rates on a continuous rise.^[1]^ In China, cardiovascular diseases account for 40.27% of total annual deaths, making it the leading cause of mortality.^[2]^ Particularly concerning is the escalating incidence of acute myocardial infarction (AMI) due to shifts in lifestyle and an aging population, posing substantial threats to patient lives and significantly reducing their quality of life, thereby imposing heavy burdens on society and families.^[3]^

AMI, a severe cardiovascular emergency, primarily results from sudden interruption or significant reduction of coronary artery blood flow. Its pathophysiological mechanisms involve coronary artery thrombosis, leading to myocardial ischemia and subsequent pathological changes at cellular and tissue levels.^[4]^ Clinically, AMI patients often present with persistent chest pain, tightness, and associated hemodynamic changes, alongside potential life-threatening arrhythmias. Without timely intervention, it can lead to heart failure, cardiogenic shock, or even cardiac arrest.^[5]^

Traditionally, management strategies post-AMI emphasized prolonged bed rest to minimize cardiac complications and risk of recurrent infarction. However, recent studies suggest that extended bed rest may not significantly improve outcomes and could instead result in adverse consequences such as muscle atrophy and thrombosis.^[6]^ This paradigm shift has prompted the medical community to reconsider post-AMI rehabilitation strategies.

With rapid advancements in rehabilitative medicine, cardiac rehabilitation has emerged as a critical component of late-phase AMI management. Extensive research confirms that well-designed cardiac rehabilitation programs not only effectively improve cardiac function but also markedly enhance quality of life, reduce readmission rates, and long-term mortality.^[7,8]^ Yet, despite its recognized clinical value, global implementation rates of cardiac rehabilitation remain disproportionately low, especially in low- to middle-income countries.^[9]^

To enhance the effectiveness and adherence of cardiac rehabilitation, researchers have proposed various theoretical models and practice frameworks. These models attempt to explain patient health behaviors from different perspectives and provide guidance for clinical interventions. However, there is currently a lack of research and comparative analyses of these theories and models.

This paper aims to comprehensively review and assess the major theories and models related to cardiac rehabilitation post-AMI, including their theoretical foundations, application methods, and clinical outcomes. By systematically analyzing the strengths and limitations of these theories and models, we aim to provide comprehensive theoretical guidance to clinicians and researchers, promoting optimization and innovation in cardiac rehabilitation practices. Additionally, this paper will explore integrating these theories and models with emerging technologies such as telemedicine and wearable devices to address current challenges in cardiac rehabilitation, thereby enhancing accessibility and effectiveness.

## 2. The concept and development of cardiac rehabilitation

The concept of cardiac rehabilitation has undergone significant evolution over the past few decades as a dynamic and expanding field. The World Health Organization (WHO) first defined cardiac rehabilitation in the 1960s as “the sum of activities that have a beneficial impact on the disease itself and the physical, psychological, and social functioning of the patient, aimed at improving quality of life and promoting a more normal restoration of social life.”^[1]^ This definition established a multidimensional and comprehensive foundation for cardiac rehabilitation, emphasizing the application of the biopsychosocial medical model.

With advancements in medical practice and research, the definition of cardiac rehabilitation has continued to refine and expand. In 2007, the American Association of Cardiovascular and Pulmonary Rehabilitation/American Heart Association (AACVPR/AHA) proposed a more comprehensive definition.^[2]^ They described cardiac rehabilitation as a multidisciplinary approach encompassing medical assessment, exercise intervention, risk factor correction, health education, and psychological counseling. This definition highlights the integrative and specialized nature of cardiac rehabilitation, underscoring the importance of multidisciplinary teamwork.

Entering the 21st century, the scope of cardiac rehabilitation has further enriched. Renowned Chinese cardiologist Professor Hu Dayi introduced the concept of the “Five Prescriptions,” namely medication prescription, exercise prescription, nutritional prescription, psychological prescription (including sleep management), and smoking cessation prescription.^[3]^ This concept expanded cardiac rehabilitation from a focus on exercise to a comprehensive, patient-centered service system, reflecting the holistic and individualized characteristics of modern cardiac rehabilitation.

Modern cardiac rehabilitation is typically divided into 3 continuous yet relatively independent stages^[4]^: in-hospital acute phase rehabilitation (stage 1), post-discharge 3 to 12 month intensive rehabilitation (stage 2), and lifelong maintenance rehabilitation at home or in the community (stage 3). This staged rehabilitation model emphasizes that cardiac rehabilitation is a continuous, long-term process requiring the collective participation of patients, healthcare teams, and the community.

Drawing from current research and practices, modern cardiac rehabilitation can be understood as a comprehensive, individualized secondary prevention strategy. It not only focuses on the restoration of cardiac function but also strives to improve overall health status, quality of life, and long-term prognosis for patients. With advancements in technology and shifts in medical paradigms, cardiac rehabilitation is evolving towards more precise, digitalized, and personalized approaches. Emerging technologies and methods such as remote cardiac rehabilitation, AI-based personalized rehabilitation programs, and wearable device-assisted continuous monitoring are reshaping the practice of cardiac rehabilitation.^[6]^

In summary, the concept of cardiac rehabilitation has evolved from simple exercise interventions to today’s multidimensional, lifecycle-oriented, and personalized comprehensive rehabilitation systems. This evolution reflects a deeper understanding in medicine of the comprehensive health needs of cardiac patients and anticipates that cardiac rehabilitation will play an increasingly important role in future cardiovascular disease management.

## 3. The significance of cardiac rehabilitation

Cardiac rehabilitation is the secondary prevention of vascular disease. It is a professional prevention and treatment system that integrates biomedicine, sports medicine, nutritional medicine, psychosomatic medicine, and behavioral medicine based on a holistic medical assessment that systematizes, structures, digitizes, and individualizes preventive management measures for cardiovascular disease through a comprehensive model of 5 core prescriptions [pharmacological prescription, exercise prescription, nutritional prescription, psychological prescription (including sleep management) and A comprehensive model of risk factor interventions through 5 core prescriptions [drug prescription, exercise prescription, nutrition prescription, psychological prescription (including sleep management) and smoking and alcohol cessation prescription] for patients with cardiovascular disease in the acute, recovery and maintenance phases and throughout life. It reduces the physical and psychological effects of heart disease, reduces the risk of infarction and sudden death, controls cardiac symptoms, stabilizes or reverses the atherosclerotic process, and improves the psychological and occupational status of patients. Studies have shown that cardiac rehabilitation improves cardiovascular outcomes and health and reduces the incidence of recurrent myocardial infarction. At the same time improve the patient’s overall physical condition, exercise, quality of life, and heart rate spectrum.^[2]^ Multiple meta-analyses showed a 20% to 25% decrease in all-cause and cardiac mortality in patients who had undergone cardiac rehabilitation in the previous years.

### 3.1. Clinical implications

The clinical significance of cardiac rehabilitation, particularly for patients with coronary artery disease who have experienced AMI, is profound. Research indicates a significant proportion of coronary artery disease patients struggle with inadequate management of risk factors. For instance, approximately 50% of patients under 70 years old have poorly controlled blood pressure, and 58% exhibit suboptimal serum cholesterol levels.^[7]^ Moreover, many patients face challenges in managing other risk factors such as smoking, obesity, and diabetes.

Cardiac rehabilitation programs have demonstrated considerable efficacy in addressing these clinical issues. Extensive research consistently shows that participation in cardiac rehabilitation can enhance exercise tolerance and physical fitness, correct modifiable risk factors – including improved control of blood pressure, lipids, and glucose levels – improve psychological well-being by reducing stress and anxiety, and significantly reduce mortality rates. Furthermore, cardiac rehabilitation is associated with improved medication adherence, reduced readmission rates, and better management of cardiovascular disease-related outcomes.

A meta-analysis revealed that participation in cardiac rehabilitation correlates with a 20% to 25% reduction in all-cause mortality and cardiac mortality over recent years.^[6]^ By addressing multiple aspects of cardiovascular health, cardiac rehabilitation offers a comprehensive approach to improving clinical outcomes for patients post-AMI and other forms of coronary artery disease (Table 1). Research indicates that cardiac rehabilitation can enhance patients’ exercise tolerance, modify risk factors such as blood pressure, lipids, and glucose, improve psychological well-being by alleviating tension, and lower mortality rates.^[10]^

**Table 1:** Comparing key cardiac rehabilitation models in their application post-AMI.

Model name	Core theoretical basis	Key components	Target population	Implementation approaches	Strengths and limitations	Strength of supporting evidence
Transtheoretical model (TTM)	Stages of change, processes of change	Precontemplation, contemplation, preparation, action, maintenance, termination	Individuals ready to change behavior	Tailored interventions based on stage of change	Emphasizes stage-based interventions; may oversimplify behavior change process	High
Information-motivation-behavioral skills model (IMB)	Information, motivation, behavioral skills	Information, motivation, behavioral skills	Individuals lacking information, motivation, or skills	Enhancing information, motivation, and skills through tailored interventions	Focuses on key determinants of behavior change; may not address environmental factors adequately	Medium to high
Self-determination theory (SDT)	Autonomy, competence, relatedness	Autonomy support, competence building, relatedness	Individuals needing support for intrinsic motivation	Creating supportive environments for autonomy and competence	Emphasizes intrinsic motivation and psychological needs; less focus on external influences	Medium
PRECEDE-PROCEED model	Planning and evaluation framework	Social, epidemiological, behavioral, and environmental assessment; policy, regulatory, and organizational components	Communities and populations requiring health interventions	Comprehensive planning from assessment to implementation	Provides a structured approach to health program planning; requires extensive data and resources	High
Family-centered empowerment model (FCEM)	Empowerment, family-centered care	Empowerment processes, family involvement	Families needing empowerment in healthcare decisions	Promoting shared decision-making and family involvement	Focuses on family dynamics and empowerment; may require strong family engagement	Medium to high
Omaha system theory (OST)	Problem classification, intervention scheme, outcome measurement	Problem classification, intervention coding, outcome measurement	Healthcare providers managing complex patient care	Standardizing data collection and intervention planning	Provides a structured framework for nursing practice; requires training and adherence	Medium
Trans theoretical model of behaviour (TTMB)	Stages of behavioral change, decisional balance	Stages of change, pros and cons of behavior change	Individuals seeking behavioral change	Using stage-specific interventions and decisional balance assessments	Focuses on decision-making and readiness for change; less on environmental factors	Medium to high

### 3.2. Economic implications

The economic impact of cardiac rehabilitation is profound, benefiting both patients and broader society in several key ways. Firstly, it reduces healthcare costs significantly by promoting healthy lifestyles and effectively managing disease progression, thereby lowering rates of readmission and complications. Research indicates that patients participating in cardiac rehabilitation experience an average reduction of 25% to 30% in healthcare expenditures over the subsequent 3 to 5 years compared to nonparticipants. Secondly, it enhances workforce productivity by improving physical function and mental health, facilitating faster return to work and maintaining higher job efficiency. This not only reduces economic losses due to absenteeism but also enhances overall societal productivity. Thirdly, it diminishes indirect costs by alleviating caregiving burdens within families and reducing reliance on social welfare expenditures. As patients’ self-care abilities improve, their dependency on family members and societal resources decreases, thereby lowering indirect economic burdens. Moreover, despite requiring initial investment, cardiac rehabilitation yields long-term economic benefits that far exceed its costs. Cost-benefit analyses suggest that every dollar invested in cardiac rehabilitation can save approximately 3 to 5 dollars in medical and social costs over the subsequent 5 years. Lastly, by decreasing emergency visits and unplanned hospital admissions, cardiac rehabilitation optimizes healthcare resource allocation and enhances overall operational efficiency of the healthcare system. In summary, cardiac rehabilitation not only saves substantial healthcare expenses for individuals but also yields significant economic benefits for healthcare insurance systems and society at large. It represents a preventive, health-promoting healthcare model that, in the long term, effectively mitigates the substantial economic burden of cardiovascular diseases, contributing towards the establishment of a more sustainable healthcare system.

### 3.3. Social relevance

The societal implications of cardiac rehabilitation are multifaceted and profound. Firstly, it significantly enhances patients’ quality of life by systematically improving physical function and providing crucial support in psychological and social dimensions. This integration facilitates better social integration and reduces the sense of isolation caused by illness. Secondly, effective cardiac rehabilitation reduces the incidence of disability resulting from cardiovascular diseases, thereby lowering societal welfare burdens and enabling more patients to sustain their work capacity and contribute to society. Studies indicate that participants in cardiac rehabilitation programs experience a 20% to 30% lower long-term disability rate compared to nonparticipants. Thirdly, these programs promote social inclusivity through group-based approaches, offering platforms for mutual support and interaction among patients. Such social engagement helps diminish disease-related discrimination and biases, fostering greater societal understanding and acceptance of cardiovascular patients. Additionally, cardiac rehabilitation initiatives not only impact patients directly but also influence their families and social circles, raising overall health awareness and promoting healthier lifestyles on a broader scale – a ripple effect that aids in cardiovascular disease prevention. Furthermore, by enhancing patients’ self-care abilities and health conditions, cardiac rehabilitation alleviates the burden on family caregivers, thereby improving family relationships and enabling better balance between work and caregiving responsibilities. Moreover, as a secondary preventive measure, cardiac rehabilitation reduces the recurrence and complications of cardiovascular diseases, thereby easing the strain on healthcare systems and promoting more equitable and efficient allocation of medical resources. Finally, these programs foster interdisciplinary collaboration among specialists including cardiologists, rehabilitation physicians, nutritionists, and psychologists, setting a precedent for managing other chronic illnesses. In summary, cardiac rehabilitation profoundly impacts society by improving patient quality of life, reducing disability rates, fostering social inclusivity, raising health awareness, easing family burdens, optimizing healthcare resource utilization, and promoting interdisciplinary cooperation, thereby contributing significantly to the development of a healthier and more inclusive society.

## 4. Theory and model of cardiac rehabilitation

Cardiac rehabilitation, as a multidisciplinary and comprehensive intervention process, has continuously evolved and improved its theoretical foundations and practical models over the past few decades. These theories and models not only provide scientific basis for cardiac rehabilitation but also guide the specific implementation of clinical practices. This section introduces several widely applied major theories and models in the field of cardiac rehabilitation.

### 4.1. Transtheoretical models

The Transtheoretical model (TTM), initially proposed by Prochaska and DiClemente in the 1980s, integrates various theories of behavior change into a comprehensive model.^[11]^ This model posits that behavior change is a gradual process rather than an instantaneous event. Central components of TTM include: stages of change (SOC), which encompass precontemplation, contemplation, preparation, action, and maintenance stages; Processes of Change (POC), describing how individuals transition between different stages; decisional balance (DB), which weighs the pros and cons of behavior change; and Self-efficacy, the individual’s confidence in their ability to successfully change behavior.

In cardiac rehabilitation, TTM is widely applied to assist patients in modifying unhealthy lifestyles such as smoking cessation, increasing exercise, and improving diet. Research indicates that interventions based on TTM significantly enhance patient compliance and rehabilitation outcomes. For instance, a study exploring the feasibility of smartphone applications in cardiac rehabilitation for coronary heart disease patients found that when applications provided recommendations aligned with TTM and other theories, patient compliance markedly improved, leading to better rehabilitation results. This study also underscores the immense potential of mobile technology in cardiac rehabilitation.

### 4.2. Information-motivation-behavioral skills (IMB) model

The information-motivation-behavioral skills (IMB) model, proposed by Fisher and Fisher in 1992, is another prominent theoretical framework widely utilized in cardiac rehabilitation to explain and predict changes in health behaviors.^[12]^ This model comprises 3 core components: Information, referring to accurate knowledge relevant to health behaviors; Motivation, encompassing individual, and social motivations, including attitudes towards behavior change and subjective norms; and behavioral skills, which denote the specific skills and self-efficacy required for executing health behaviors.

The IMB model is appreciated for its clarity, operational ease, and comprehensive consideration of multiple key factors influencing behavior change. It finds applications not only in cardiac rehabilitation but also in various other chronic disease management domains. For instance, studies by Li Shumin et al^[13]^ in oncology have applied health education based on the IMB model to nasopharyngeal cancer radiotherapy patients, resulting in improved self-management skills and rehabilitation compliance. Additionally, the IMB model has been effectively employed in patient education for conditions such as tuberculosis, post-hip replacement surgery in the elderly, pre-cardiac conditions, bladder ostomy, hemodialysis, stroke, and schizophrenia. These applications have been proven beneficial in enhancing patient lifestyles, quality of life, and adherence.

**Figure 1.**
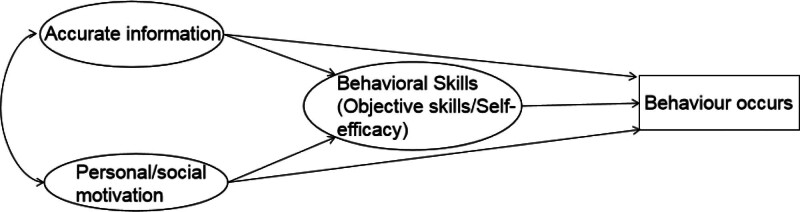
IMB model framework diagram.

In cardiac rehabilitation specifically, the IMB model is instrumental in designing comprehensive intervention strategies. For example, in studies focusing on coronary heart disease patients, interventions based on the IMB model significantly enhanced aerobic endurance, muscle strength, daily life management, cognitive symptom management, and disease management scores (see Fig. 1).

### 4.3. Self-determination theory

Self-determination theory (SDT), developed by Deci and Ryan in the 1980s, is a theory based on human psychological growth and developmental motivation.^[14]^ SDT posits that the satisfaction of basic psychological needs and autonomous or intrinsic motivation is closely linked to personal enthusiasm and performance. Among numerous motivational theories, SDT has found extensive application in the field of cardiac rehabilitation.

SDT categorizes motivation into autonomous motivation (including identification, integration, and intrinsic motivation) and controlled motivation (including external regulation and introjected regulation), based on the degree of autonomy. The theory connects positive or negative behaviors with specific social contextual factors, suggesting that these factors influence motivation by affecting individuals’ basic psychological needs (autonomy, competence, and relatedness), thereby leading to positive or negative behavioral outcomes.

In the realm of cardiac rehabilitation, SDT has been employed to explore psychological well-being and behavioral regulation. A study implemented a 12-month rehabilitation supervision program for all participants, assessing them before intervention and 6 months post-intervention withdrawal. The study measured patients’ psychological needs, behavioral regulations, self-esteem, anxiety, depression, and other indicators. Analysis revealed that increased autonomy satisfaction predicted positive behavioral changes, while improvements in competence and relatedness satisfaction corresponded to enhanced performance and increased well-being.^[10]^ This research not only elucidates psychological changes occurring during cardiac rehabilitation but also underscores the importance of rehabilitation programs and long-term physical activities in meeting patients’ psychological needs, enhancing motivational factors, and improving patient happiness. These findings provide a critical theoretical foundation and practical guidance for designing more effective cardiac rehabilitation programs.

### 4.4. The PRECEDE-PROCEDE model

The PRECEDE-PROCEED model was first proposed by American epidemiologists and educational experts, including Green et al, in the 1980s.^[15]^ This model has found extensive application in the management of chronic diseases, although its effectiveness in certain health domains remains incompletely validated.^[16,14]^ Nevertheless, it has demonstrated significant efficacy in alleviating depressive symptoms and enhancing quality of life.

This model comprehensively analyzes health factors from various perspectives including geographical, environmental, organizational, managerial, educational, behavioral, and societal aspects. It addresses specific health issues through a systematic approach: assessing needs, formulating intervention strategies, implementing interventions, evaluating outcomes, ultimately aiming to address patient health concerns and promote overall well-being.

Wei et al^[17]^ conducted a controlled trial exploring the impact of a health education intervention based on the PRECEDE-PROCEED model on improving quality of life for heart failure patients. The intervention group participated in a 2-month health education program, meeting weekly for 60 to 90 minutes. Three months post-intervention, participants completed four questionnaires. Results indicated that the health education program developed under this model effectively mitigated depressive symptoms, enhanced self-monitoring capabilities, and improved quality of life.

The PRECEDE-PROCEED model has been applied in diverse international research contexts. Future studies could further integrate it with cognitive differences, behavioral habits, cultural characteristics, and traditional practices specific to different countries, thereby devising more precise and personalized rehabilitation intervention plans. Such integration aims to better meet the personalized rehabilitation needs of various patient types, fully leveraging the model’s strengths and effects.

### 4.5. Family-centered empowerment model

The family-centered empowerment model (FCEM) is designed to enhance the quality of life for chronic disease patients by empowering families through increased empowerment levels and the elimination of health and well-being barriers.^[18,19]^ At its core, this model involves healthcare professionals assisting family members in gaining caregiving knowledge, skills, and resources to actively manage their lives, thereby improving the overall quality of life for both patients and their families.

The fundamental principle of FCEM is empowerment. Throughout this process, healthcare professionals collaborate closely with primary caregivers to develop personalized care plans aimed at addressing specific challenges encountered during caregiving, thus enhancing their caregiving abilities and preparedness. This approach not only focuses on the patient themselves but also recognizes the role and needs of the family unit as a whole.

In recent years, FCEM has been widely adopted by international scholars in the management of various chronic diseases, gradually becoming an effective measure to enhance the quality of life for chronic disease patients and their families. To validate the model’s effectiveness in the field of cardiovascular disease, a randomized controlled trial examined the therapeutic effects of FCEM on the psychological status of myocardial infarction patients.

The study divided the intervention process into 4 stages: assessment stage: researchers comprehensively assessed the physiological and psychological conditions of patients through group meetings. Education stage: Following AMI, patient expectations were assessed, and learning and experience-sharing took place among groups of 3 to 4 members. Implementation stage: Formative and summative assessments were conducted on patients, with continuous monitoring of intervention effects. Evaluation stage: Comprehensive assessment of intervention outcomes. The research findings indicate significant improvements in anxiety and stress symptoms among patients who received FCEM intervention. Importantly, the intervention group showed marked enhancements in overall health levels, including physical functioning, social adaptation abilities, and psychological well-being.

### 4.6. Omaha system theory

The Omaha system is 1 of the standardized nursing language systems recognized by the American Nurses Association (ANA).^[17]^ It consists of 3 core components: the problem classification scheme, intervention scheme, and problem rating scale for outcomes. Its primary application lies in systematically documenting patient health issues and tailoring nursing interventions based on individual patient needs, thereby promoting standardized and quantifiable clinical nursing practices.

Currently, the Omaha System finds widespread use in health management and has been adopted and endorsed by healthcare systems in multiple countries. Its feasibility in the Chinese context has been validated through introduction and application studies. However, it is important to note that the application of the Omaha system should be appropriately adjusted according to disease characteristics and cultural backgrounds.

In the context of patients undergoing percutaneous coronary intervention (PCI), application results have shown that nursing interventions based on the Omaha System can improve patient behaviors and quality of life. Liu et al^[20]^ implemented continuous nursing interventions based on the Omaha system for PCI patients, aiming to study its effects on medication adherence, quality of life, and prognosis for coronary heart disease patients. The study results indicated significant improvements in medication adherence and a notable reduction in adverse event rates after 9 months of continuous care.

The application of the Omaha system theory not only reduces the incidence of cardiovascular adverse events post-PCI and improves patient prognosis but also significantly enhances patient satisfaction with nursing services. These findings underscore the potential value of the Omaha system in cardiac rehabilitation. However, it is important to note the study’s limitation of a small sample size. To further validate the specific effects of the Omaha System, multi-center comparative studies are recommended to obtain more comprehensive and reliable evidence.

### 4.7. Trans theoretical model of behaviour

The trans theoretical model of behavior (TTM) is a comprehensive theory of behavior change comprising 4 core components: SOC, POC, self-efficacy, and DB. The SOC form the central organizing structure of the model, encompassing precontemplation, contemplation, preparation, action, and maintenance.

What sets TTM apart is its integration of 19 different behavior change frameworks, providing a systematic approach to assessing intervention development. This integration makes TTM a practical scientific framework for intervention design. However, due to its emphasis on using the best available evidence, applying TTM often requires a significant time investment and a deep understanding of the model by the team.

Nevertheless, TTM has shown significant success in intervention development, particularly in enhancing motivation and capabilities among healthcare professionals. This has been well demonstrated in the field of cardiac rehabilitation.

A randomized pilot study conducted by Zhang et al^[8]^ explored the impact of behavior change theory-based interventions on post-PCI patients’ quality of life. The study confirmed that behavior change theory-based interventions effectively improve patients’ quality of life and lifestyles in cardiac rehabilitation management post-PCI for coronary heart disease patients.

Further supporting these findings, Zhou Yingying et al^[21]^ found that behavior change theory-based cardiac rehabilitation interventions not only promote behavior change and reduce coronary artery disease risk factors but also enhance general self-efficacy, improve quality of life, and foster overall health in PCI patients. These research findings highlight the potential and value of TTM in cardiac rehabilitation, particularly in promoting long-term behavior change and improving patient outcomes.

## 5. Summary of shortcomings and outlook

Cardiac rehabilitation is a highly specialized field distinct from cardiology and cardiac surgery. It requires collaboration among various professionals including cardiovascular physicians, cardiac rehabilitation specialists, physiotherapists, and psychologists to ensure the quality and effectiveness of implementation. Key factors for the smooth development of cardiac rehabilitation clinics include clinicians’ research, understanding, and emphasis on specialized knowledge in cardiac rehabilitation, as well as hospital leadership’s attention, overall planning, and coordination of relevant departments.

Compared to developed countries, China currently has room for improvement in its cardiac rehabilitation rate. Additionally, low participation and poor compliance in cardiac rehabilitation are prominent global issues, including in China. Therefore, future academic efforts should focus on conducting more research and exploration in this field, generating higher-level evidence in evidence-based medicine. This will provide patients with more comprehensive, scientific, systematic, and practical guidance for cardiac rehabilitation, thereby achieving greater rehabilitation benefits.

Looking ahead, theories, models, and approaches can integrate with emerging technologies, new equipment, and information technology to help patients overcome barriers and difficulties in participating in cardiac rehabilitation from multiple perspectives and levels. Introducing new treatment methods has the potential to provide patients with greater flexibility, making the rehabilitation process more enjoyable and interactive. Intelligent rehabilitation exercise methods that align with modern habits and preferences can enhance people’s enthusiasm and compliance with rehabilitation. These innovations may reduce constraints related to environment, location, equipment, methods, and personnel when patients engage in cardiac rehabilitation at home or in the community. Future research directions and goals may aim to minimize constraints related to time, place, environment, equipment, and personnel for cardiac rehabilitation, making it more accessible and convenient.

Suggestions for future research should focus on several aspects: developing and validating cardiac rehabilitation models and intervention strategies suitable for the Chinese cultural context, exploring effective methods to increase participation and compliance in cardiac rehabilitation, especially tailored to Chinese patients. Assessing the application effectiveness of new technologies in cardiac rehabilitation, including remote rehabilitation and mobile health applications. Strengthening interdisciplinary cooperation to integrate the strengths of different professions and provide more comprehensive cardiac rehabilitation services. Conducting long-term follow-up studies to assess the long-term effects and cost-effectiveness of different cardiac rehabilitation strategies.

Through these efforts, we hope to provide more personalized, efficient, and convenient rehabilitation services for cardiac patients in the future, ultimately improving patient prognosis and quality of life.

## Author contributions

**Conceptualization:** Jia Liu, Qiannan Zhao, Chen Lu.

**Data curation:** Jia Liu, Qiannan Zhao.

**Formal analysis:** Jia Liu, Qiannan Zhao.

**Funding acquisition:** Qiannan Zhao.

**Investigation:** Qiannan Zhao, Yi Zheng.

**Methodology:** Qianqian Shen, Xiaoqin Meng, Chen Lu.

**Project administration:** Qianqian Shen, Xiaoqin Meng.

**Resources:** Xiaoqin Meng.

**Software:** Yi Zheng.

**Supervision:** Yeping Zheng.

**Validation:** Chen Lu, Yeping Zheng.

**Visualization:** Chen Lu, Yeping Zheng.

**Writing – original draft:** Jia Liu, Qiannan Zhao, Qianqian Shen, Xiaoqin Meng, Yi Zheng, Chen Lu.

**Writing – review & editing:** Yeping Zheng.
